# DPP-based polymers with linear/branch side chain for organic field-effect transistors

**DOI:** 10.3389/fchem.2022.1008807

**Published:** 2022-09-13

**Authors:** Daohai Zhang, Dongxu Liang, Liang Gu, Jianhui Li, Haichang Zhang

**Affiliations:** ^1^ School of Chemical Engineering of Guizhou Minzu University, Guiyang, China; ^2^ Key Laboratory of Rubber-Plastics of Ministry of Education/Shandong Province (QUST), School of Polymer Science and Engineering, Qingdao University of Science and Technology, Qingdao, China

**Keywords:** organic semiconductor, conjugated (conducting) polymers, polymer, donor–acceptor conjugated polymers, charge transport mobilities

## Abstract

For polymer semiconductors, the packing ability and molecular weight of polymers play a very critical role in their optoelectronic properties and carrier transport properties. In this work, two polymers, named linear and branch, are designed and synthesized with donor–acceptor (D-A) structure, based on diketopyrrolopyrrole as an electron acceptor and carbazole as an electron donor, and applied these two polymers in organic field-effect transistors. Linear and branch have similar conjugated backbones but different molecular weights and alkyl chains. The effects of molecular weight and molecular aggregation ability on the carrier transfer efficiency are investigated. As a result, linear exhibits better aggregation ability, but due to its smaller molecular weight than branch molecule, the hole transfer efficiency of linear (1.1 × 10^−2^ cm^2^ V ^−1^ s^−1^) is slightly lower than that of branch (2.3 × 10^−2^ cm^2^ V ^−1^ s^−1^). This work proves that molecular weight is more important than molecular aggregation ability when designing organic field-effect transistors for polymer semiconductors.

## Introduction

In the past few decades, organic conjugated polymers have been widely used in the field of field-effect transistors due to their flexible molecular structures and good solubility (Guo et al., 2013; Moon et al., 2018; Michael, 2021). In the late 1980s, the first organic field-effect tube made of polythiophene was developed for the first time (Cui et al., 2018; Zou et al., 2019). This kicked off the development of organic field-effect transistors (OFETs). After decades of development, OFETs have made great progress (Pan et al., 2018; Ran et al., 2020; Raveendran and Namboothiry, 2020).

At the beginning of this century, a wide variety of OFET materials sprung up like mushrooms after a rain. In the process of improving device performance, a large number of conjugated polymer materials have been developed. The molecular design of high-performance conjugated polymers is based on the concepts of molecular orbital energetics and crystal engineering, enabling efficient control of frontier orbital energy levels and π orbital overlap (Nahid et al., 2016; Moon et al., 2018; Qu et al., 2021; Ozdemir et al., 2017; Ozdemir et al., 2020). Therefore, in the past research, there are a lot of strategies to explore the structure of polymer main chain and side chain, such as the main chain π-conjugated orbital extension, main chain planarity improvement, and side chain and main chain hydrogen bonding engineering (Wang et al., 2018; Cheon et al., 2022; Kang et al., 2022). It was found that polymers with branched side chains can effectively increase the solubility of polymers. However, branched chains might affect the *π*-π stacking properties of molecules, which is not conducive to the transfer of carriers; thus, the application of branch alkyl chains in conjugated polymers remains to be further investigated (Liu et al., 2017; Pie et al., 2017).

In this work, we designed a series of DPP-CZ-based conjugated polymers with octane and 2,2,4-trimethylpentane focusing on the effects of branched vs. linear and molecular weight on polymer device performance. The aggregation and crystallization properties of the materials were investigated by XRD. Although the electrochemical properties of the polymers were very similar, their device properties showed some differences due to different molecular weights.

## Result and discussion

### Synthesis and properties of DPP-based polymers

**FIGURE 1:**
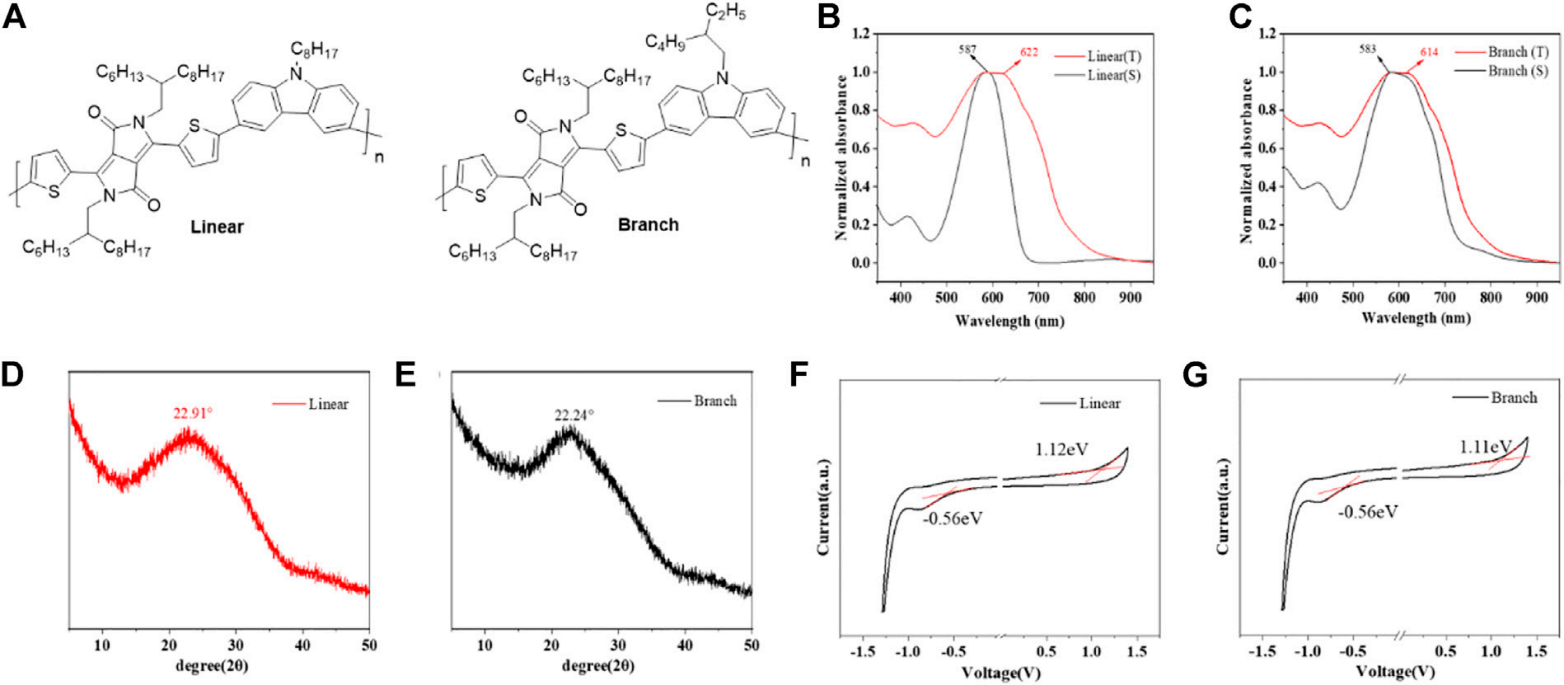
**(A)** Chemical structures of both linear and branch polymers. **(B,C)** UV/vis absorption spectra of both polymers in solution and thin film state. **(D, E)** XRD spectra of both polymers. **(F,G)** Cyclic voltammograms of both polymers (spin-coated thin films on the ITO glass). Electrolyte: 0.1 M tetrabutylammonium hexafluorophosphate in acetonitrile. Potential calculated versus ferrocene. Scan rate: 100 mV s^−1^. Temperature: room temperature.

Two polymers, named linear and branch (Figure 1A), are synthesized by the Suzuki coupling reaction through diketopyrrolopyrrole (DPP)-based monomers as electron acceptor units and carbazole groups with different alkyl chains as electron donor units (Supplementary Schemes S5, S6). In the process of synthesizing the polymer, the molecular weight of the polymer is controlled by changing the length of the polymerization time. The main chains of the two polymers are fixed so as to compare the molecular weight and the alkyl side chain of the polymer in the construction of high-performance organic field-effect transistors. The molecular weight (*M*_*w*)_ values of linear and branch polymers were 12 and 22 kDa, and the PDI is 1.7 and 1.9, respectively.

### Optical and electrochemical properties

Figures 1B and C show the UV-Vis absorption spectra of the two polymers in solution and film state, and their specific parameters are listed in Table 1. Since they have similar main chain structures, their UV absorptions also show very similar curves, but due to the difference in the alkyl chains, in contrast, linear has a red-shift of 15 nm in the absorption maximum from solution to thin film samples, while branch is 11 nm, indicating that linear molecules have slightly stronger aggregation ability, which was considered to be able to effectively improve the carrier transfer efficiency in past studies. The onset absorption of both polymer films starts at 775 nm, based on which the optical band gaps are calculated to be 1.60 eV for branch and 1.59 eV for linear, respectively. In order to further verify the effect of the alkyl chain on molecular aggregation ability and *π*-π stacking ability, XRD was tested as shown in Figures 1D and 3. Both molecules showed intermolecular co-facial *π*-π stacking peaks, located at 22.91° and 22.24°, corresponding to the d-spacing of 0.387 and 0.40 nm for linear and branch polymers, respectively.

**TABLE 1:** Optical and electrochemical properties of branch and linear.

	λ_abs,max_		HOMO^a^ [eV]	LUMO^a^ [eV]	E_g_ ^b^/Eopt g^c^ [eV]
	Solution	Thin film			
Branch	583	614	-5.92	-4.24	1.67/1.60
Linear	587	622	-5.91	-4.24	1.68/1.59

a HOMO level was obtained by CV measurement: −E_HOMO_ = E_onset(ox)_ + 4.8eV; E (LUMO) = −e [E_red_ _onset_+ 4.8] eV, where E_onset(ox)_ is the onset potential for the oxidation versus ferrocene.

b Electrochemical band gap.

c Optical band gap Eopt **g** was determined at the absorption onset of the material in the solution phase (Eopt **g** = 1240/λ_abs,onset_ eV).

As shown in Figures 1F and G, the two polymers exhibited very similar electrochemical profiles due to their identical backbone structural units. Both polymers showed similar oxidation onset potentials around 1.11–1.12 eV, based on which the highest occupied molecular orbital (HOMO) energy levels were calculated to be −5.92 eV and −5.91 eV for branch and linear polymers, respectively. Similar electrochemical profiles suggest that the two polymers can exhibit similar hole-injection abilities after being assembled into OFETs. This also proves that the difference between the two devices mainly comes from the difference in molecular weight and side chain. The electrochemical band gap is 1.67 eV for branch and 1.68 eV for linear, whose value is similar to the optical ones.

### OFET

**FIGURE 2:**
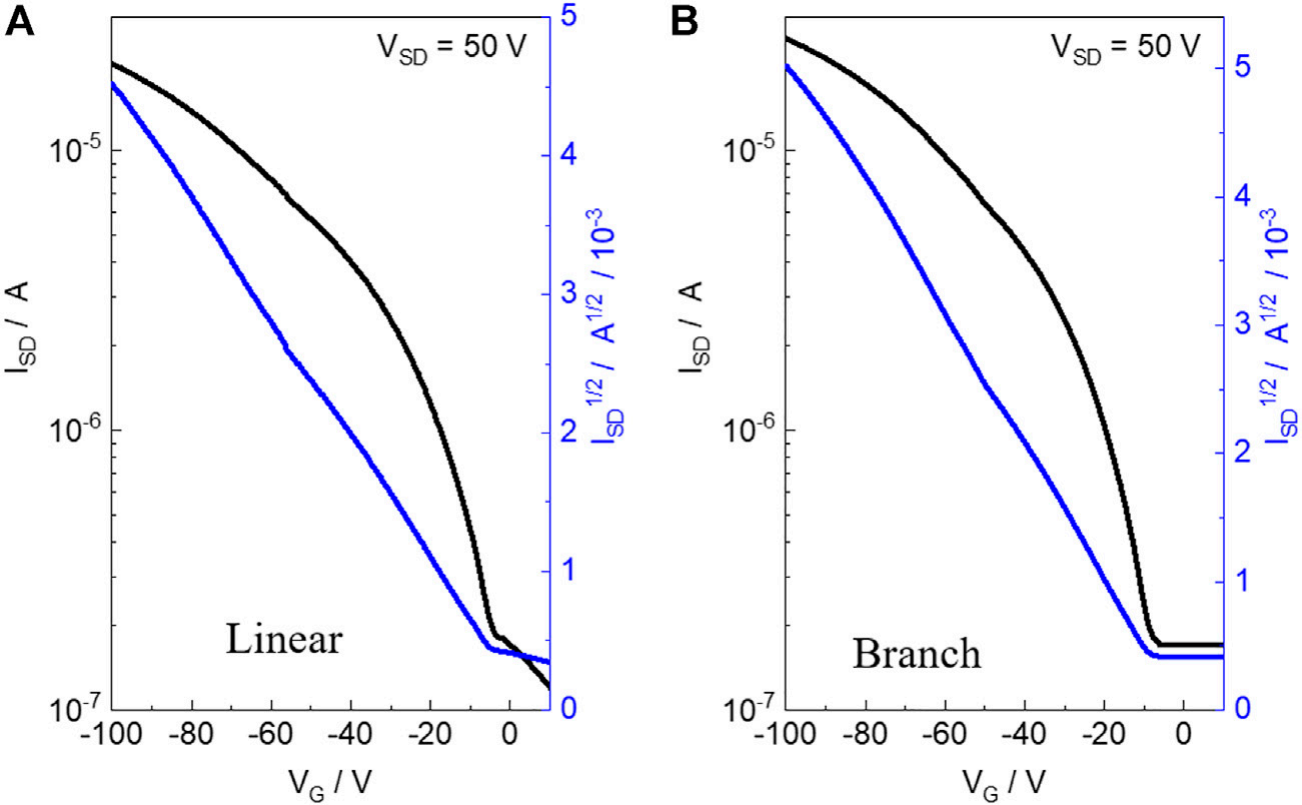
Transfer characteristics of the OFET devices based on linear polymer **(A)** and branch polymer **(B)**.

The charge transport properties of the two polymers were evaluated by fabricating the OFET devices of the materials with a bottom-gate and bottom-contact (BGBC) configuration on an n-type silicon wafer using a layer of 300 nm SiO_2_ as the dielectric material. The linear and branch polymer-based devices were fabricated *via* directly spin-casting the polymer solution in chloroform onto the OTS-treated silicon wafer with pre-patterned gold source and drain electrodes, which were further measured under vacuum conditions. The detailed device fabrication and testing procedures are described in the Supporting Information. As shown in Figure 2, both polymers exhibited p-type properties with the highest hole mobility (μ_h_) estimated to be 1.1 × 10^−2^ cm^2^ V ^−1^ s^−1^ (average μ_h_: 9 × 10^−3^ cm^2^ V ^−1^ s^−1^) and 2.3 × 10^−2^ cm^2^ V ^−1^ s^−1^ (average μ_h_: 1.9 × 10^−3^ cm^2^ V ^−1^ s^−1^) for linear and branch obtained from eight different devices, respectively. Both polymers present a similar polymer backbone. In addition, compared to the branch polymer, the linear one exhibits improved aggregation and *π*-π stacking. Thus, high charge mobility is expected for the linear polymer. However, in this work, we found that the branch one presents a double value for hole transport mobility when compared to the linear one. This indicates that to obtain a high-performance semiconductor, molecular weight is more important than molecular aggregation ability for the molecular design concept. The current ratio of both OFET devices is in the range of 10^2^–10^3^.

## Conclusion

In this work, two polymers, named linear and branch, are synthesized and characterized. These two polymers present almost the same polymer backbone and the chemical structures, except the side alkyl chains are linear or branch. The XRD as well as the optical absorption studies indicate that the linear polymer exhibits stronger aggregation than the branch one. Normally, strong aggregation is beneficial for charge transport between individual molecules. Thus, the linear polymer with high charge transport mobility is expected. However, the GPC measurement shows that the branch polymer has a high molecular weight, which might be due to the fact that the branch polymer exhibits improved solubility compared to linear ones. As a result, linear polymer presents lower hole transport mobility than the branch one, which indicates that molecular weight is more important than molecular aggregation ability when designing organic field-effect transistors for polymer semiconductors. This work paves the way for future molecular design to fabricate high-performing semiconductor polymer materials for a wide range of applications.

## Data Availability

The original contributions presented in the study are included in the article/Supplementary Material; further inquiries can be directed to the corresponding author.
