# Supplementary material for: DPP-based polymers with linear/branch side chain for organic field-effect transistors
Source: Front Chem. 2022 Sep 13;10:1008807. doi: 10.3389/fchem.2022.1008807 (PMC9514454; doi:10.3389/fchem.2022.1008807)
Supplement: Supplementary file 1 [file DataSheet1.docx]

**Supporting Information**

**DPP-based polymers with linear/branch side chain for organic field-effect transistors**

**Daohai Zhang,^1a^ Dongxu Liang, ^1b^ Liang Gu, ^b^ Jianhui Li, ^b^ Haichang Zhang ^b*^**

**^a^** School of Chemical Engineering of Guizhou Minzu University, Guizhou,Guiyang 550025, China

^b^Key Laboratory of Rubber-Plastics of Ministry of Education/Shandong Province (QUST), School of Polymer Science and Engineering, Qingdao University of Science and Technology, 53-Zhengzhou Road, Qingdao 266042, P. R. China

^1^ Daohai Zhang and Rui Li contribute equally for this work.

* Corresponding author: Haichang Zhang: haichangzhang@hotmail.com

**Contents**

**1. Experimental Procedures**

[**1.** **Experimental Section** 3](#_Toc103515796)

[*1.1 Materials* 3](#_Toc103515797)

[*1.2 Synthesis* 3](#_Toc103515798)

[*1.2.1 synthesis route of L-CZ* 3](#_Toc103515799)

[*1.2.2 synthesis route of B-CZ* 3](#_Toc103515800)

[*1.2.3 synthesis route of L-Bo* 4](#_Toc103515801)

[*1.2.4 synthesis route of B-Bo* 4](#_Toc103515802)

[*1.2.5 synthesis route of Linear* 4](#_Toc103515803)

[*1.2.6 synthesis route of Branch* 5](#_Toc103515804)

[*1.3 Characterization for Linear and Branch* 5](#_Toc103515805)

[*1.3.1 Structure characterization* 5](#_Toc103515806)

[*1.3.2 Electrochemical properties measurements* 5](#_Toc103515807)

[*1.3.3 UV/vis absorption measurements* 5](#_Toc103515808)

[*1.3.4 Thin film structures characterization* 5](#_Toc103515809)

[*1.3.5 OFET devices fabrication* 5](#_Toc103515810)

[**2. Results** 6](#_Toc103515811)

[*2.1 NMR of compound L-BO* 6](#_Toc103515812)

[*2.2* *NMR of compound B-BO* 7](#_Toc103515813)

[*2.3 NMR of compound LINEAR* 7](#_Toc103515814)

[*2.4 NMR of Branch* 8](#_Toc103515815)

[**3. References** 8](#_Toc103515816)

# **Experimental Section**

## *1.1 Materials*

All reagents were purchased from commercial sources and used without further purification unless otherwise noted. 1-Bromooctane, 2-Ethylhexyl bromide, 3,6-Dibromocarbazole, NaOH, Sodium borohydride, KOAc, PdCl_2_[DPPF], K_2_CO_3_, Pd(PPh_3_)_4_, Bis(pinacolato)diboron, and the solvents were obtained from Energy Chemical.DPP-C16 were obtained from hyperchemical.

## *1.2 Synthesis*

### *1.2.1 synthesis route of L-CZ*

Scheme S1 synthesis route of **L-CZ**

In a clean single-necked flask, add Br-CZ (3.25g, 10mmol), 1-bromooctane (4.4g, 23mmol), NaOH (2.4g, 60mmol), and DMSO (30ml), under nitrogen protection, After stirring at room temperature for 12 hours, extract with water and dichloromethane, dry the organic phase with anhydrous magnesium sulfate, remove excess solvent by distillation under reduced pressure, and settle in methanol to obtain L-CZ (4.11g, yield: 93.8%) as white crystals, no further purification was required.

### *1.2.2 synthesis route of B-CZ*

Scheme S 2 synthesis route of **B-CZ**

In a clean single-necked flask was added Br-CZ (3.25g, 10mmol), 2-Ethylhexyl bromide (4.4g, 23mmol), NaOH (2.4g, 60mmol), and DMSO (50m;), under nitrogen protection conditions, after stirring at room temperature for 12h, extract with water and dichloride, dry the organic phase with anhydrous magnesium sulfate, remove excess solvent by vacuum distillation, and settle in methanol to obtain B-CZ (3.9g, yield: 89.2%) as a colorless oily liquid, no further purification was required.

### *1.2.3 synthesis route of L-Bo*

Scheme S 3 synthesis route of **L-Bo**

In a clean single-necked flask was added L-CZ (0.2g, 0.45mmol), KOAc (0.1347g, 1.35mmol), pinacol biboronate (0.348g, 1.35mmol) followed by PdCl2[DPPF] (0.01g, 0.014mmol), under nitrogen protection, the system was heated to 100 ° C and reacted for 18 hours, extracted with water and dichloride, the organic phase was dried with anhydrous magnesium sulfate, and the excess solvent was removed by distillation under reduced pressure. The crude product was purified by column chromatography (silica gel, EA: petroleum ether = 1:8) to afford compound L-BO(0.184 g, yield: 76.2 %) as a white solid. ^1^H NMR (500 MHz, CHCl_3_-d_1_) δ ppm: 8.66 (s, 2H), 7.89-7.90 (d, 2H), 7.38-7.39(d, 2H),4.28-4.31 (t, 2H), 1.83(s, 2H),1.38(s, 24H),1.21-1.34(m, 10H) 0.83-0.86(m,3H).

### *1.2.4 synthesis route of B-Bo*

Scheme S 4 synthesis route of **B-Bo**

In a clean single-necked flask was added B-CZ (0.2g, 0.45mmol), KOAc (0.1347g, 1.35mmol), biboronate pinacol ester (0.348g, 1.35mmol) followed by PdCl2[DPPF] (0.01g, 0.014mmol), under nitrogen protection, the system was heated to 100 ° C and reacted for 18 hours, extracted with water and dichloride, the organic phase was dried with anhydrous magnesium sulfate, and the excess solvent was removed by distillation under reduced pressure. The crude product was purified by column chromatography (silica gel, EA: petroleum ether = 1:8) to afford compound B-BO (0.206 g, yield: 85.1 %) as a white solid. ^1^H NMR (500 MHz, CHCl_3_-d_1_) δ ppm: 8.66 (s, 2H), 7.87-7.89 (d, 2H), 7.35-7.37(d, 2H), 4.14-4.17 (t, 2H), 2.03(s, 1H),1.25(s, 32H), 0.82-0.89(m,6H).

### *1.2.5 synthesis route of Linear*

Scheme S 5 synthesis route of **Linear**

In a clean single-neck flask, L-BO (0.10557g, 0.2mmol), DPP-C_16_ (0.18140g, 0.2mmol), K_2_CO_3_ (1.38g, 2M/L), toluene (15ml), H_2_O (5ml) were added, after 3 times of nitrogen replacement, [PPh_3_]_4_Pd (0.0069g, 0.0006mmol) was added, then after 3 times of nitrogen replacement, the system was heated to 95 ℃, and after 24 hours of reaction, extraction with dichloromethane and deionized water, the organic phase was dried with anhydrous magnesium sulfate, the excess organic solvent was removed, and the crude product was dropped into methanol, followed by filtration to obtain Linear (170 mg, 82%).^1^H NMR (500 MHz, CHCl_3_-d_1_) δ ppm: 8.99-9.01 (d), 8.50-8.59 (m), 8.38(s), 7.87-7.89(d), 7.73-7.74(d), 7.28-7.50(m), 7.14-7.23(m), 5.23(s), 4.23-4.26(m), 4.03-4.07(m), 3.893-3.91(d),1.72-1.90(m), 1.16-1.35(m), 0.71-0.80(m).

### *1.2.6 synthesis route of Branch*

Scheme S 6 synthesis route of **Branch**

In a clean single-neck flask, B-BO (0.10557g, 0.2mmol), DPP-C_16_ (0.18140g, 0.2mmol), K_2_CO_3_ (1.38g, 2M/L), toluene (15ml), H_2_O (5ml) were added, after 3 times of nitrogen replacement, [PPh_3_]_4_Pd (0.0069g, 0.0006mmol) was added, then after 3 times of nitrogen replacement, the system was heated to 95℃, and after 48 hours of reaction, extraction with dichloromethane and deionized water, the organic phase was dried with anhydrous magnesium sulfate, the excess organic solvent was removed, and the crude product was dropped into methanol, followed by filtration to obtain Branch (174 mg, 84%).^1^H NMR (500 MHz, CHCl_3_-d_1_) δ ppm: 8.93-8.94(d), 8.51-8.52(d), 7.74-7.76(d), 7.48-7.49(d), 7.36-7.38(d), 7.15(d), 4.74-4.79(m), 4.02-4.04(m), 3.84-3.91(m), 0.72-0.79(m), 0.81-0.85(m).

## *1.3 Characterization for Linear and Branch*

## *1.3.1 Structure characterization*

NMR spectra were obtained using a Mercury 500 spectrometer. Elemental analysis was performed on a Carlo Erba 1106 Elemental Analyzer.

## *1.3.2 Electrochemical properties measurements*

Cyclic voltammetry (CV) measurements were performed with a BAS 100 W Bioanalytical Systems, using a glass carbon disk (Φ = 3 mm) as the working electrode, a platinum wire as the auxiliary electrode with a porous ceramic wick, and Ag/Ag^+^ as the reference electrode, standardized for the redox couple ferricinium/ferrocene. All solutions were purged with a nitrogen stream for 10 min before measurement. The procedure was performed at room temperature and a nitrogen atmosphere was maintained during the measurements.

## *1.3.3 UV/vis absorption measurements*

UV/vis absorption spectra were recorded using a dual-beam grating Agilent Cary 5000 absorption spectrometer. The thin film UV/Vis absorption spectra of these two molecules were measured by using spin-coated thin film (7 mg/mL molecules in chloroform spin-coated on quartz glass substrate, rotation speed: 1200 rpm). Solution is prepared by the materials with [concentration](http://www.baidu.com/link?url=4gtF6_zERMn3MamImQa0YXEQ3sI703Ty_OYHYYU_PvSJjMzQxbkj4JFc8CfP1wR0E1kko1HadlHbxEnAx0Nsc1qUhgJ24y-mfmfBQzBPxQm) of 1×10^-4^ mg /mL.

## *1.3.4 Thin film structures characterization*

Thin film X-ray diffraction (XRD) experiments working at 3 KW were performed on a Powder X-ray Diffractometry (INCA Energy, Oxford Instruments). The films were prepared by spin-coasting of small molecules’ solution (7 mg / ml in chloroform).

## *1.3.5 OFET devices fabrication*

Bottom gate, top contact thin-film field effect transistors configuration was used to evaluate the small molecules semiconductors. Highly doped Si wafer with 300 nm thermal oxide (Silicon Quest International) was used as the substrate, where the doped silicon layer was used as back gate (G) and the oxide as the dielectric materials (*C_i_*, capacitance of 10 nF·cm^–2^). After cleaning the substrate with Piranha (H_2_SO_4_/H_2_O_2_ = 3/1), DI water and acetone, plasma clean (10 min, PDC-001), the substrate was put in OTS solution (5 % in toluene) at room temperature for over nigh in Ar-filled glove box. Subsequently the source (S) and drain (D) electrode pairs were electron-beam evaporated on the surface of the silicon wafer by using Au target (with a thickness of 50 nm) through a mask. The semiconductor layer was deposited though spin-coated by the polymer Branch and Linear’ solution (8 mg/mL in chloroform). The polymer transistor devices were further treatment by thermal annealing at 50 ^o^C for 0.5 h in order to make sure all the chloroform evaporation. The TFT device has a channel length (*L*) of 30 µm with a channel width (*W*) of 1 mm. The devices were characterized in air using a Hewlett Packard 4155A semiconductor analyzer. The field effect mobility was calculated using *I*_SD_ = (*W*/2*L*)·*µC*_i_ (*V*_G_ – *V*_T_)^2^, where *L* and *W* are the channel length and width; *C_i_* is the capacitance of gate oxide, *V_SD_* is voltage between source and drain electrodes; *I_SD_* is the current between source and drain electrodes; *V_G_* is the gate voltage.

# **2. Results**

## *2.1 NMR of compound L-BO*


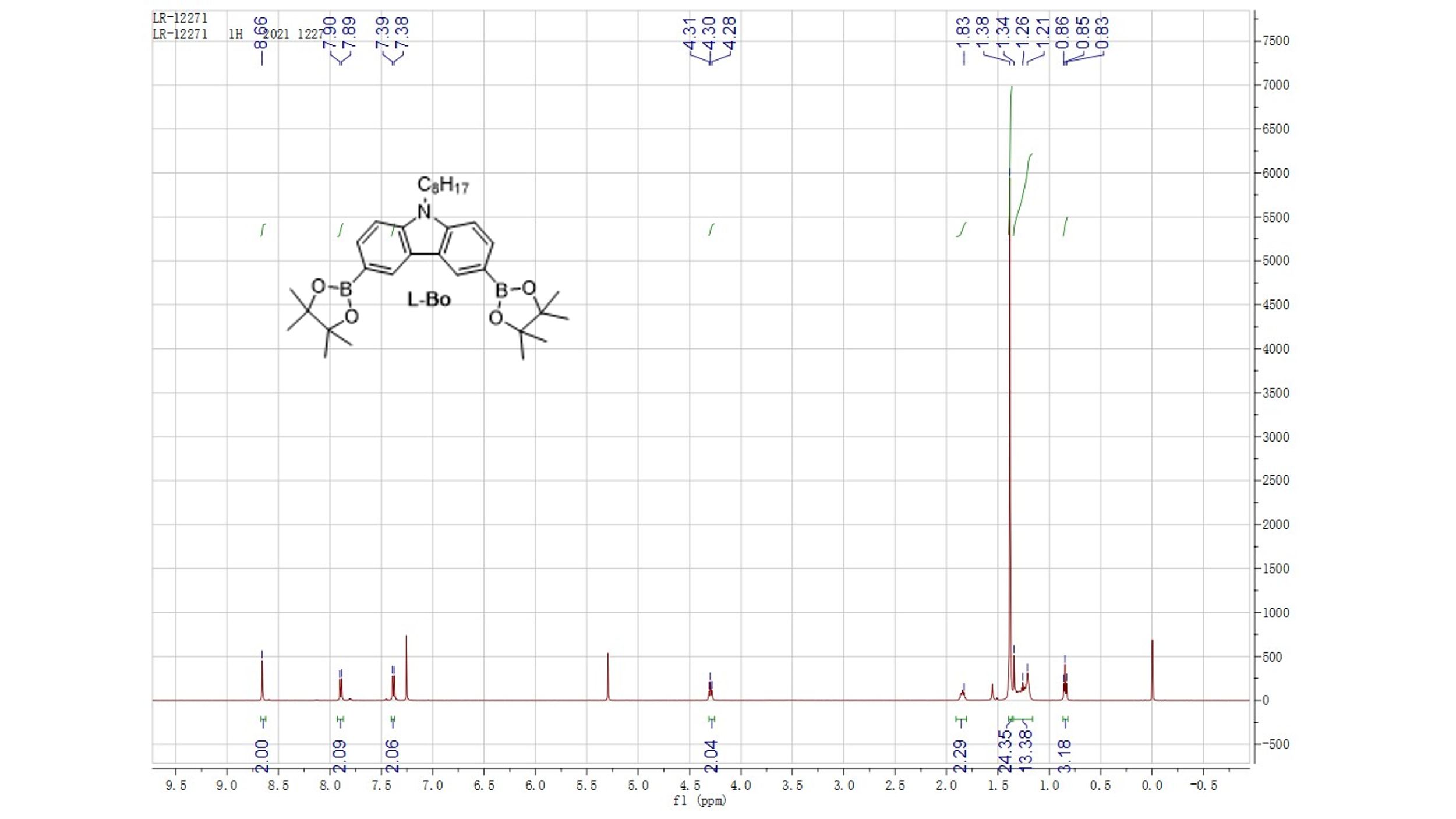


**Figure S1.** ^1^H NMR spectra of compound L-BO

## *NMR of compound B-BO*


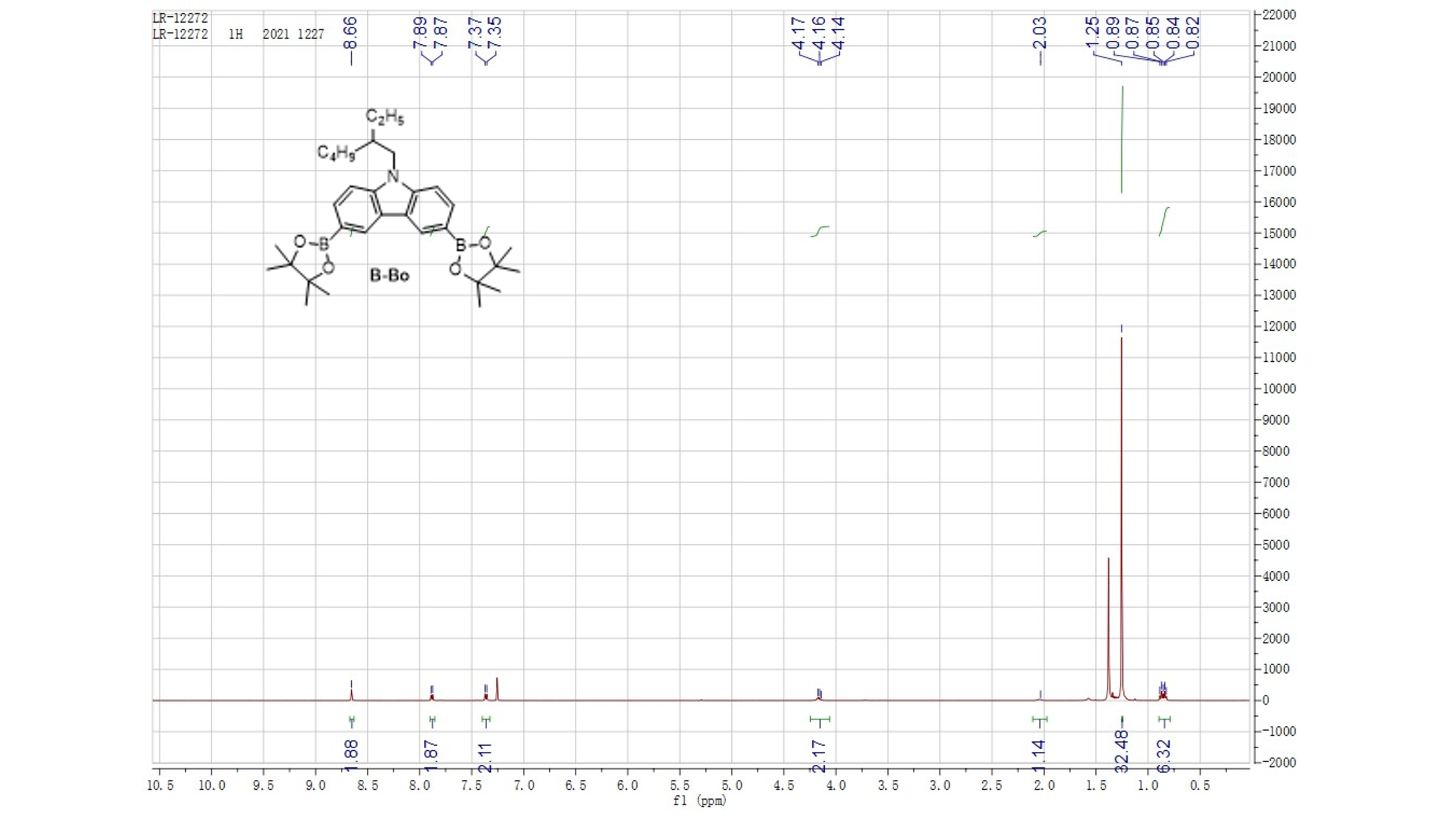


**Figure S2.** ^1^H NMR and ^13^C NMR spectra of compound B-BO

## *2.3 NMR of compound Linear*


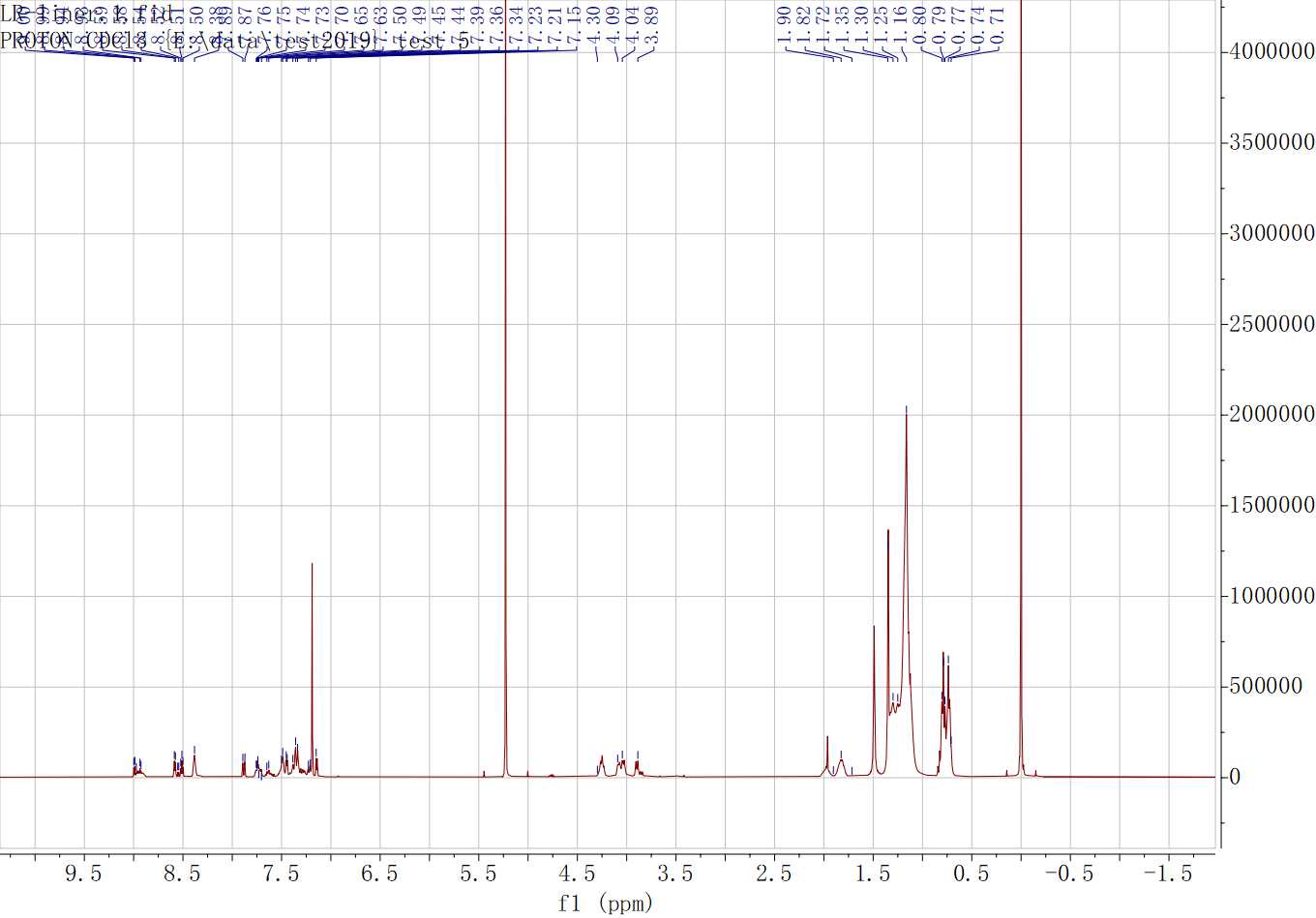


**Figure S3.** ^1^H NMR spectra of compound Linear

## *2.4 NMR of Branch*


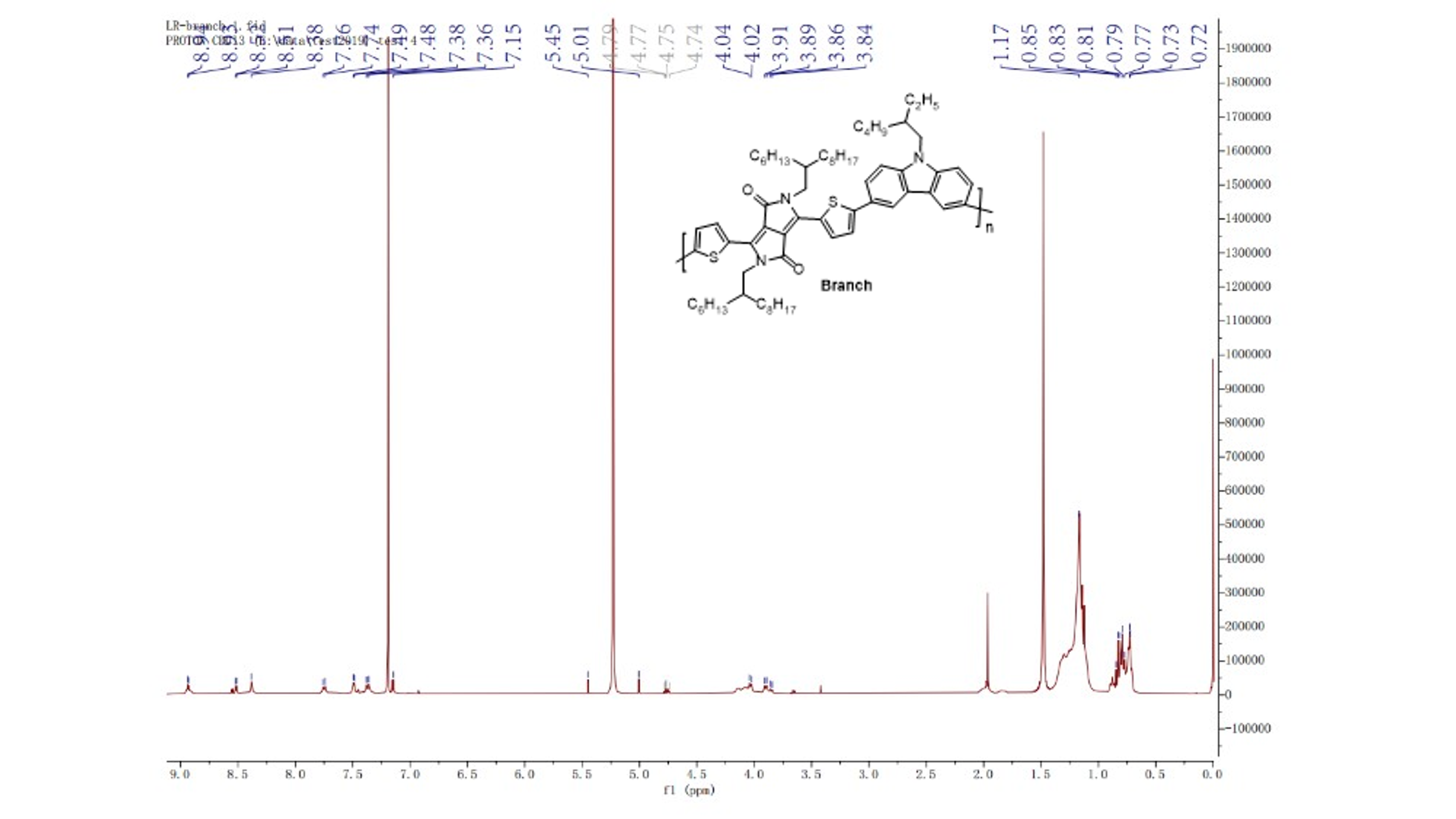


**Figure S4.** ^1^H NMR spectra of Branch

# **3. References**

[1] Li S, He Z, Yu J, Chen S, Zhong A, Wu H, Zhong C, Qin J, Li Z. 2,3-bis(5-Hexylthiophen-2-yl)-6,7-bis(octyloxy)-5,8-di(thiophen-2-yl) quinoxaline: A good construction block with adjustable role in the donor-π-acceptor system for bulk-heterojunction solar cells. J Polym Sci Pol Chem 2012;50:2819-28
